# TRAF3 can interact with GMEB1 and modulate its anti-apoptotic function

**DOI:** 10.1186/s40709-020-00117-2

**Published:** 2020-05-29

**Authors:** George Kotsaris, Despoina Kerselidou, Dimitrios Koutsoubaris, Elena Constantinou, George Malamas, Dimitrios A. Garyfallos, Eudoxia G. Ηatzivassiliou

**Affiliations:** 1 Department of Genetics, Development and Molecular Biology, School of Biology, Aristotle University of Thessaloniki, University Campus, 54124 Thessaloniki, Macedonia Greece; 2 Department of Medicine, School of Health Sciences, Aristotle University of Thessaloniki, University Campus, 54124 Thessaloniki, Macedonia Greece; 3 Present Address: Institute for Chemistry and Biochemistry, Freie Universität Berlin, Thielallee 63, 14195 Berlin, Germany; 4 Present Address: Université de Liège, Place du 20-Août, 7 B, 4000 Liège, Belgium; 5 Present Address: Wellcome Trust Sanger Institute, Genome Campus, Hinxton, Cambridgeshire, CB10 1SA UK

**Keywords:** Signal transduction, Apoptosis, TRAF3, GMEB1, Protein–protein interaction, TNF

## Abstract

**Background:**

Members of Tumor Necrosis Factor (TNF) Receptor-Associated Factors (TRAFs) family interact with the cytoplasmic tails of TNF receptor family members to mediate signal transduction processes. TRAF3 has a major immunomodulatory function and TRAF3 deficiency has been linked to malignancies, such as multiple myeloma and lymphoid defects. In order to characterize the molecular mechanisms of TRAF3 signaling, the yeast two-hybrid system was used to identify proteins that interact with TRAF3.

**Results:**

The yeast two-hybrid screen of a human B cell cDNA library with TRAF3 as bait, identified Glucocorticoid Modulatory Element-Binding Protein 1 (GMEB1) as a TRAF3-interacting protein. Previous studies indicated that GMEB1 functions as a potent inhibitor of caspase activation and apoptosis. The interaction of TRAF3 and GMEB1 proteins was confirmed in mammalian cells lines, using immunoprecipitation assays. The RING and TRAF-C domains of TRAF3 were not essential for this interaction. The overexpression of TRAF3 protein enhanced the anti-apoptotic function of GMEB1 in HeLa cells. On the other hand, downregulation of TRAF3 by RNA interference decreased significantly the ability of GMEB1 to inhibit apoptosis. In addition, LMP1(1–231), a truncated form of the EBV oncoprotein LMP1, that can interact and oligomerize with TRAF3, was also able to cooperate with GMEB1, in order to inhibit apoptosis.

**Conclusions:**

Our protein-interaction experiments demonstrated that TRAF3 can interact with GMEB1, which is an inhibitor of apoptosis. In addition, cell viability assays showed that overexpression of TRAF3 enhanced the anti-apoptotic activity of GMEB1, supporting a regulatory role of TRAF3 in GMEB1-mediated inhibition of apoptosis. Better understanding of the molecular mechanism of TRAF3 function will improve diagnostics and targeted therapeutic approaches for TRAF3-associated disorders.

## Background

TRAF3 belongs to the family of TNF-Receptor-Associated Factors (TRAFs) [1, 2]. The highly conserved carboxy-terminal region of these proteins (TRAF-C domain) interacts with the cytoplasmic region of TNFRs. The amino-terminal region contains a RING and zinc-finger domains, which are required for TRAF-mediated signaling, that includes the activation of the canonical Nuclear Factor-κB (NF-κB) and specific MAPK signaling pathways. RING-containing TRAF proteins can act as E3-ubiquitin ligases, that can catalyze the formation of K63-linked polyubiquitin chains, which serve as signaling scaffolds [3, 4]. In the absence of relevant extracellular signals, TRAF3 cooperates with TRAF2 and mediates the degradation of NIK kinase to prevent the activation of the non-canonical NF-κB pathway [5]. Signaling processes that activate the non-canonical NF-κB pathway, induce the degradation or sequestration of TRAF3. TRAF3 can also mediate the activation of interferon response pathways, which are involved in antiviral activities [5, 6]. Mutations that inhibit the function of TRAF3 lead to constitutively active NF-κB in patients with multiple myeloma [7]. Interestingly, the human papilloma virus (HPV) positive head and neck squamous cell carcinoma (HNSCC) tumors have much higher frequency (~ 22%) of deep deletions and truncations of TRAF3, compared to the HPV negative HNSCC tumors [8, 9]. In addition, TRAF3 deficiency can lead to Herpes Simplex Virus (HSV) encephalitis [10]. Furthermore, TRAF3 inactivating mutations have been linked to multiple types of B-cell lymphomas [11–13]. Consequently, better characterization of the molecular mechanisms of TRAF3 function will improve diagnostics and lead to new therapeutic approaches for TRAF3 associated disorders.

In order to elucidate the TRAF3-mediated signaling mechanisms, we performed a yeast two-hybrid screen for the identification of TRAF3-interacting proteins. The present study focuses on the identification of the GMEB1 protein (Glucocorticoid Modulatory Element-Binding Protein 1) as a TRAF3-interacting protein and the functional characterization of this interaction. Initially, it was reported that GMEB1 is a transcriptional factor that modulates the activity of glucocorticoid receptor [14]. Additionally, it was shown that GMEB1 interacts with caspases 8 and 9 and inhibits apoptosis of neuronal cells after stress [15, 16]. We have shown that overexpression of TRAF3 protein enhanced the anti-apoptotic role of GMEB1.

## Results

### TRAF3 can interact with GMEB1

**Fig. 1:**
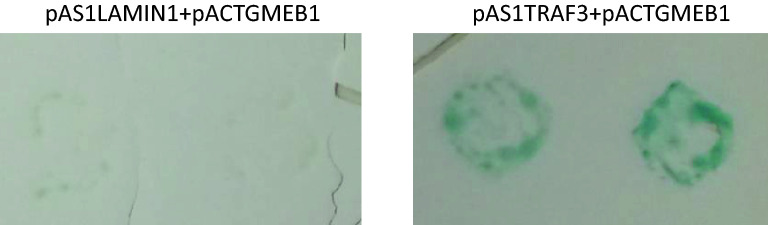
Identification of GMEB1 as a TRAF3-interacting protein by the yeast two hybrid system. Yeast colonies co-transformed with a plasmid expressing a fusion of GMEB1 to the Gal4 transactivating domain (pACTGMEB1) and a plasmid expressing a Gal4 DNA-binding domain fusion with TRAF3(12–568) (pAS1TRAF3) or LAMIN1 (pAS1LAMIN1) were tested for their ability to transactivate a Gal4-dependent β-galactosidase reporter gene using a filter lift assay

**Fig. 2:**
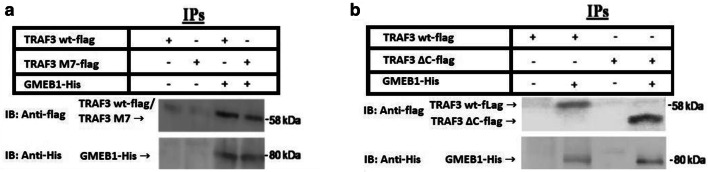
Co-immunoprecipitation of TRAF3 and GMEB1 in mammalian cells. HEK293 cells were cotransfected with vectors expressing a His-tagged GMEB1 protein (GMEB1-His) (1 μg) together with (**a**) a FLAG-tagged TRAF3 protein (TRAF3 wt-flag) (0.5 μg) or a FLAG-tagged mutant TRAF3 M7 (TRAF3 M7-flag) (0.5 μg), defective in the RING domain. **b** a truncated TRAF3 (TRAF3ΔC-flag) (0.5 μg), which is lacking the TRAF-C domain. 24 h post transfection, the cell extracts were subjected to immunoprecipitation using the Ni–NTA beads (Qiagen), which bind to GMEB1-His. The immunoprecipitated complex (IP) was analyzed by SDS-PAGE on a 7.5% gel and subjected to immunoblot analysis with an anti-His rabbit polyclonal antibody recognizing GMEB1 (His probe) (80 kDa) or with an anti-flag mouse monoclonal antibody recognizing both TRAF3 wt (58 kDa) and TRAF3 M7 (58 kDa)

In an effort to understand the molecular mechanisms of TRAF3 function, a yeast two hybrid screen was performed. In this screen, a GAL4 fusion of human TRAF3 was used as bait to screen a cDNA library made from a human lymphoblastoid cell line [17, 18]. One of the clones identified codes for GMEB1 (Fig. 1). To confirm the ability of TRAF3 to interact with GMEB1, it was examined whether the two proteins could be co-immunoprecipitated in mammalian cells. For this purpose, a FLAG-tagged TRAF3 expressing (TRAF3 wt flag) and a His-tagged GMEB1 expressing vector (GMEB1-His) were co-transfected in HEK 293 FT cells. Upon immunoprecipitation of GMEB1-His, TRAF3 wt-flag could be co-immunoprecipitated, as shown in Fig. 2a. Additionally, we examined whether the interaction of TRAF3 with GMEB1 was dependent on the integrity of the RING domain of TRAF3 protein. A plasmid expressing a FLAG-tagged TRAF3 mutated isoform (TRAF3 M7-flag) with a defective RING domain was constructed for this purpose. In TRAF3 M7-flag expressing plasmid, Cys-53 and Cys-56 codons were changed to Ala codons. As shown in Fig. 2a, the RING domain was not essential for the interaction of TRAF3 with GMEB1, since TRAF3 M7-flag could be co-immunoprecipitated with GMEB1-His. In addition, the TRAF-C domain of TRAF3 was not necessary for the interaction between TRAF3 and GMEB1, since GMEB1-His could be co-immunoprecipitated with a truncated TRAF3 (TRAF3ΔC), which is lacking the TRAF-C domain (Fig. 2b).

### Expression of TRAF3 can modulate the antiapoptotic function of GMEB1

**Fig. 3:**
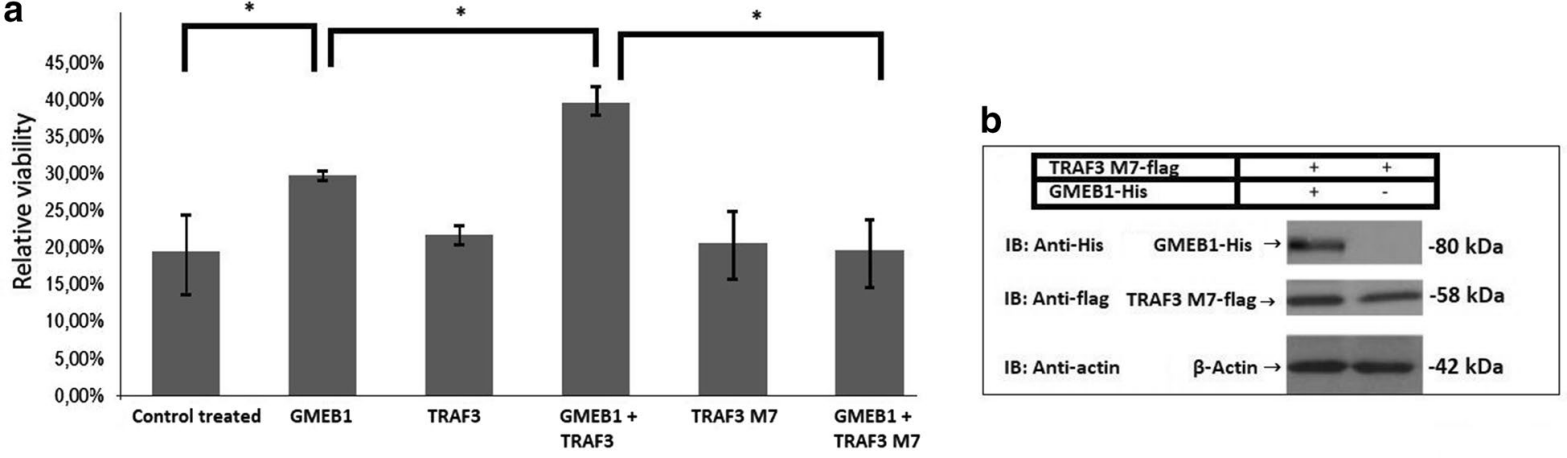
The effect of TRAF3 on GMEB1-mediated antiapoptotic function. **a** Assessment of viability of HeLa cells treated by CHX and TNFα, using MTT assay. TRAF3 or TRAF3 M7 (0.5 μg) and GMEB1 (1 μg) expressing vectors were cotransfected to HeLa cells. 20 h postransfection, HeLa cells were treated with CHX and TNFα for 24 h, in order to induce apoptosis. Viability is calculated as the ratio of O.D. sample/O.D. control untreated * 100. The data are presented as the mean ± standard error (± SEM) of three independent repetitions. Statistical evaluation of the differences between the values was performed by Student’s t-test. Statistically significant differences are indicated by brackets and an asterisk (*p* < 0.05). **b** Immunoblot analysis. Total cell lysates from HeLa cells transfected with the vectors expressing the indicated proteins were analyzed by SDS-PAGE on a 8% gel. The detection of a catalytically inactive mutant (TRAF3 M7-flag) and the GMEB1-His was performed using the antibodies that are reported in “Methods” section. β-actin was used as a loading control

GMEB1 has been shown to inhibit apoptosis of neuronal cells after stress, by interacting with caspases 8 and 9 [15, 16]. It is conceivable that TRAF3 could affect the antiapoptotic function of GMEB1 by virtue of its ability to interact with GMEB1. In order to test this hypothesis, the effect of exogenous expression of TRAF3 on the antiapoptotic function of GMEB1 was examined in HeLa cells. As expected, expression of GMEB1 inhibited the apoptosis of HeLa cells, which was induced by the addition of cycloheximide (20 μg ml^−1^) and TNFα (10 ng ml^−1^) (Fig. 3a). Interestingly, co-expression of GMEB1 and TRAF3 further enhanced the antiapoptotic function of GMEB1 (Fig. 3a). Expression of TRAF3 alone did not alter the survival of HeLa cells in the presence of cycloheximide and TNFα, indicating that the effect of TRAF3 is exerted only in the presence of GMEB1. The expression of the transfected proteins was confirmed by immunoblot analysis (Fig. 3b).

In order to investigate whether the RING domain of TRAF3 is involved in the modulation of the antiapoptotic function of GMEB1, the mutated isoform of TRAF3, TRAF3 M7-flag, with a defective RING domain was overexpressed in HeLa cells, in combination with GMEB1-His. The expression of the transfected proteins was confirmed by immunoblot analysis (Fig. 3b) and the viability of HeLa cells in the presence of cycloheximide and TNFα was tested as described above. Surprisingly, the mutant TRAF3 M7-flag did not affect the antiapoptotic activity of GMEB1, despite the fact that it could interact with the GMEB1 to the same extent as wt TRAF3 does (Fig. 3a). Taken together, these findings indicate that elevated expression of TRAF3 can modulate the antiapoptotic function of GMEB1, in a manner that depends on the integrity of the RING domain.

### TRAF3 gene silencing abolishes the antiapoptotic function of GMEB1 

**Fig. 4:**
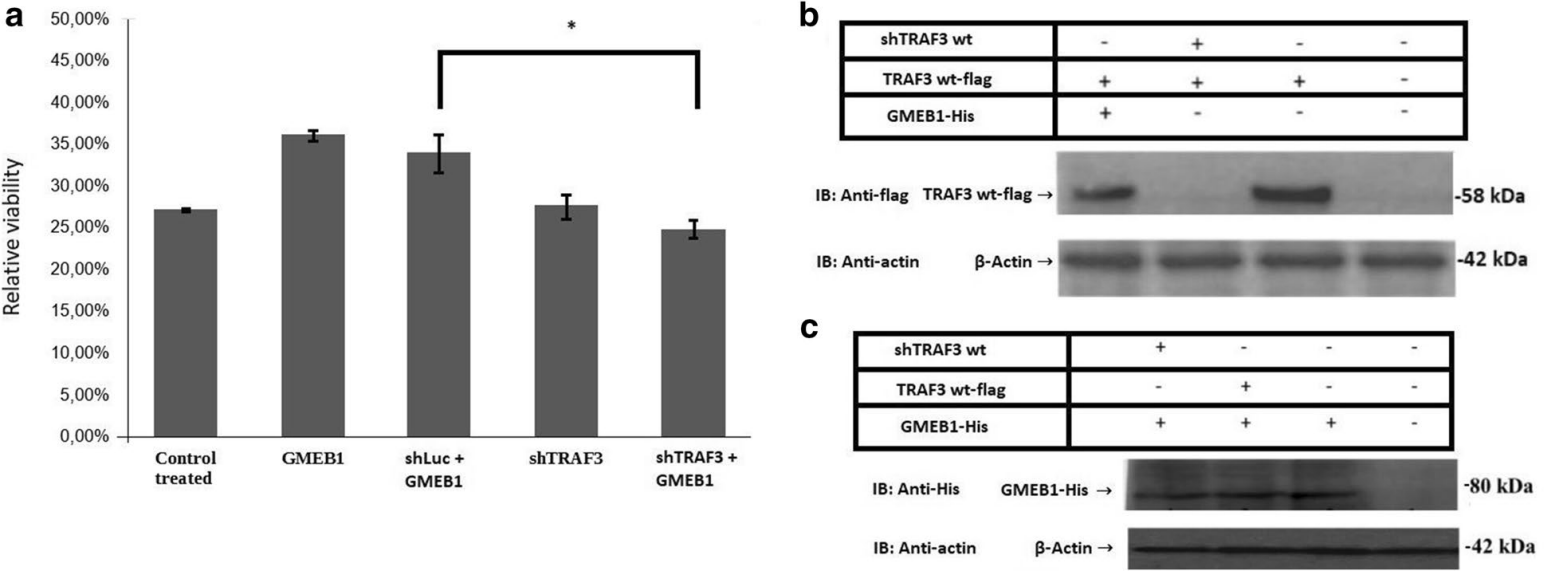
Traf3 gene silencing abolishes the antiapoptotic function of GMEB1. **a** Assessment of viability of HeLa cells, treated by CHX and TNF-α, using MTT assay. A shRNA-expression vector targeting TRAF3 (shRNATRAF3) (0.5 μg), a control shRNA-expressing vector (shRNA luc) (0.5 μg) and GMEB1-His (1 μg) were cotransfected to HeLa cells. 20 h postransfection, HeLa cells were treated with CHX and TNF-α for 24 h, in order to induce apoptosis. Viability is calculated as the ratio of O.D. sample/O.D. control untreated * 100. The data are presented as the mean ± standard error (± SEM) of three independent repetitions. Statistical evaluation of the differences between the values was performed by Student’s t-test. Statistically significant differences are indicated by brackets and an asterisk (*p* < 0.05). **b**, **c** Immunoblot analysis. Total cell lysates from HeLa cells transfected with shRNATRAF3 or control shRNAluc, and GMEB1-His were analyzed by SDS-PAGE on an 8% gel. The detection of the indicated proteins was performed using the antibodies reported in “Methods” section. β-actin was used as a loading control

To further investigate the involvement of *TRAF3* in the ability of GMEB1 to inhibit apoptosis, it was determined whether the endogenous TRAF3 is required for the antiapoptotic activity of GMEB1. RNA interference experiments were performed, using a vector expressing a TRAF3-targeting shRNA (shRNATRAF3). For this purpose, HeLa cells were co-transfected with the plasmids indicated in Fig. 4a, followed by MTT viability assays, 24 h after the addition of cycloheximide (20 μg ml^−1^) and TNFα (10 ng ml^−1^). Reduction of TRAF3 expression was successful, as it was shown by immunoblot analysis (Fig. 4b). Our results indicate that *TRAF3* gene downregulation compromises the antiapoptotic function of GMEB1.

### Investigation of the functional interaction between LMP1 and GMEB1 proteins

**Fig. 5:**
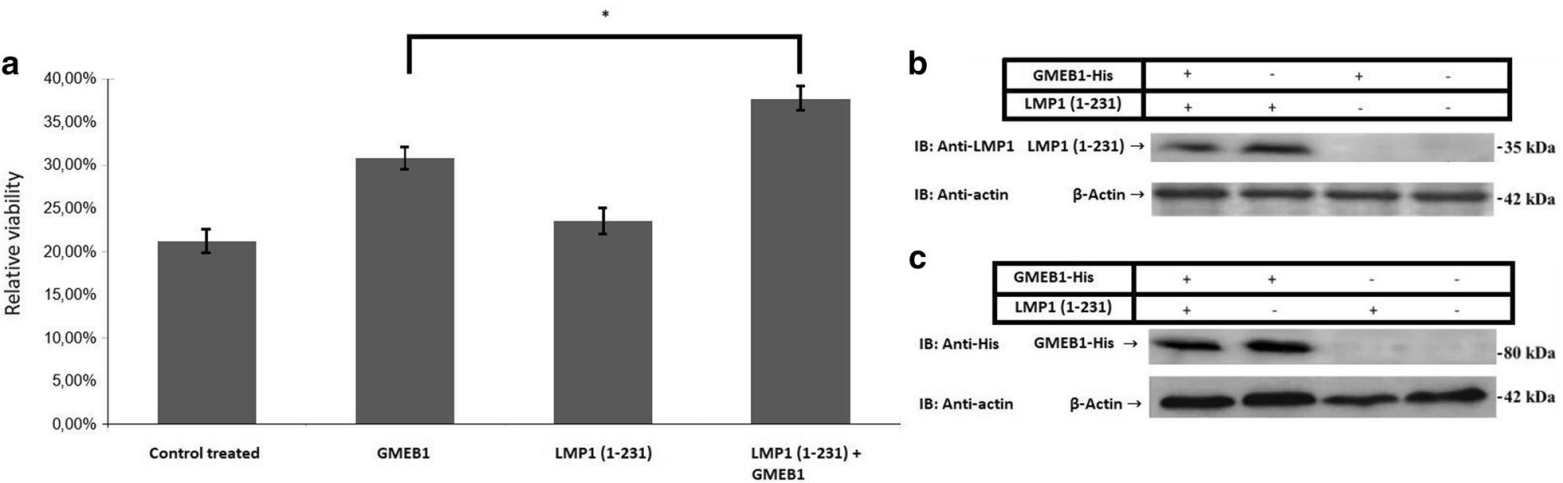
The effect of LMP(1–231) on GMEB1-mediated antiapoptotic function. **a** Assessment of viability of HeLa cells, treated by CHX and TNFα, using MTT assay. LMP(1–231) (0.5 μg) expression vector and GMEB1-His (1 μg) were co-transfected to HeLa cells. 20 h postransfection, HeLa cells were treated with CHX and TNF-α for 24 h, in order to induce apoptosis. Viability is calculated as the ratio of O.D. sample/O.D. control untreated * 100. The data are presented as the mean ± standard error (± SEM) of three independent repetitions. Statistical evaluation of the differences between the values was performed by Student’s t-test. Statistically significant differences are indicated by brackets and an asterisk (*p* < 0.05). **b** Immunoblot analysis. Total cell lysates from HeLa cells transfected with LMP(1–231) and GMEB1-His were analyzed by SDS-PAGE on an 8% gel. The detection of the indicated proteins was performed using the antibodies reported in “Methods” section. β-actin was used as a loading control

LMP1 is an oncoprotein expressed by the Epstein Barr virus (EBV) and has been shown to interact with and lead to oligomerization of TRAF3 [1]. Given the ability of TRAF3 to enhance the antiapoptotic effect of GMEB1, it is conceivable that LMP1 may modulate the antiapoptotic function of GMEB1 through its interaction with TRAF3. LMP1 contains two signaling domains (CTAR1 and CTAR2) in its cytoplasmic carboxy-terminal region, only one of which (CTAR1) interacts with TRAF3. To test specifically the role of TRAF3-interacting LMP1 domain in the antiapoptotic effect of GMEB1, a truncated form of LMP1 expressing plasmid [LMP1 (1–231)], containing only the TRAF3-interacting signaling domain, was introduced in HeLa cells, together with GMEB1 expression plasmid (Fig. 5). LMP1 (1–231) was able to enhance the antiapoptotic function of GMEB1. Our results suggest that LMP1 may modulate the antiapoptotic activity of GMEB1 by virtue of its ability to interact with TRAF3.

## Discussion

In an effort to characterize the molecular mechanisms that underlie TRAF3 signaling, GMEB1 was identified as a new TRAF3-interacting protein, using the yeast two-hybrid assay. GMEB1 has been shown to function as a caspase inhibitor, preventing neuronal apoptosis [15, 16]. Thus, we have further pursued the function of the TRAF3-GMEB1 heterodimer. First, the interaction of TRAF3 with GMEB1 was confirmed in mammalian cells, using in vitro co-immunoprecipitation assays. Notably, exogenous expression of TRAF3 enhanced the antiapoptotic function of GMEB1, as shown by MTT viability assays in HeLa cells, co-expressing TRAF3 and GMEB1. Interestingly, the TRAF3 M7 mutant, which has a defective RING domain, did not augment the antiapoptotic function of GMEB1, despite the fact that it could interact with GMEB1 to the same extent as wt TRAF3 does. Taken together, these results indicate that TRAF3 can modulate the antiapoptotic function of GMEB1, in a manner that depends on the integrity of the RING domain. In support of this notion, it was shown that downregulation of TRAF3 decreased significantly the antiapoptotic effect of GMEB1. Finally, our findings show that the EBV oncoprotein LMP1, which leads to oligomerization of TRAF3, is also able to cooperate with GMEB1 in order to inhibit apoptosis.

In our experiments, conditions that induce oligomerization of TRAF3, such as its overexpression or LMP1 expression [19], enhance the GMEB1 antiapoptotic effect. TRAF3 belongs to a family of RING-dependent ubiquitin ligases that can be activated by oligomerization [20]. Therefore, our data support a model in which activation of the ubiquitin ligase activity of TRAF3 positively regulates the GMEB1 inhibitory function on apoptosis. The mechanism by which TRAF3 affects GMEB1 activity may be through the ubiquitination of GMEB1 or a regulator of GMEB1. This possibility is supported by the inability of TRAF3 M7 mutant, to enhance the antiapoptotic activity of GMEB1. TRAF3 can mediate K63-linked ubiquitination and this activity depends on its RING domain [20]. The TRAF3 M7 mutant lacks the ubiquitin ligase activity. K63-linked polyubiquitination does not lead to protein degradation; rather, it can modulate the activity of the target protein by modifying its structure [21]. Alternatively, it can serve as a scaffold for the formation of multiprotein complexes that can activate enzymes and propagate signaling processes [22]. Therefore, it is possible that TRAF3-mediated K63-polyubiquitination of GMEB1 or an associated protein facilitates the formation of a caspase-inhibitory complex by GMEB1.

It is conceivable that ligand-induced activation of receptors, which engage TRAF3, can increase the inhibitory activity of GMEB1 towards caspases. This mechanism may contribute to the antiapoptotic activity of TNFR family members, such as CD40, LTβR and BAFFR [23–25]. The EBV oncoprotein LMP1 mimics activated receptors of the TNFR family [23, 26] and our experiments suggest that the antiapoptotic function of LMP1 can be mediated, at least in part, by TRAF3-mediated activation of GMEB1.

## Conclusions

Our study has identified a novel interaction between TRAF3 and GMEB1, which is an inhibitor of apoptosis. In addition, it was shown that overexpression of TRAF3 enhanced the anti-apoptotic function of GMEB1, supporting a regulatory role of TRAF3 in GMEB1-mediated inhibition of apoptosis. Our findings suggest a novel mechanism for the regulation of TNFα induced apoptosis, which may have significant implications in diagnosis and personalized treatment of pathologies associated with TRAF3.

## Methods

### Plasmids

Plasmids expressing LMP1 and TRAF3 were previously described [1]. Plasmid TRAF3 M7-flag is expressing a double mutant of TRAF3, where Cys-53 and Cys-56 codons were changed to Ala codons in the RING domain of TRAF3 wt-flag [27].

The vector expressing GMEB1 was a kind gift of Dr. Simons SS Jr. A shRNA-expression vector (shRNATraf3) that targets TRAF3 mRNA for RNA interference was constructed as follows: forward primer B: 5′-GATCCCCCAAGAGAGCATCGTTAAAGATAATTCAAGAGATTATCTTTAACGATGCTCTCTTGTTTTTA-3′ and reverse primer B: 5′-AGCTTAAAAACAAGAGAGCATCGTTAAAGATAATCTCTTGAATTATCTTTAACGATGCTCTCTTGGGG-3′ were phosphorylated with polynucleotide kinase and ligated to pHebo vector [28], digested with *Hpa*I and *Xho*I restriction endonucleases. A control shRNA-expression vector that targets luciferase mRNA was constructed as above, using the forward primer 5′-GATCCCCAACGTACGCGGAATACTTCGATTTTCAAGAGAAATCGAAGTATTCCGCGTACGTTTTTTTA-3′ and reverse primer 5′-AGCTTAAAAAAACGTACGCGGAATACTTCGATTTCTCTTGAAAATCGAAGTATTCCGCGTACGTTGGG-3′.

### Yeast Two-Hybrid Screening

Yeast Two-Hybrid Screening was performed using TRAF3 as a bait, as it was previously described [1, 17]. Briefly, TRAF3 codons 12-568 were fused to the DNA binding domain of GAL4 [G4DBDLAP1(12-568)] [1] and were transformed into yeast strain Y190. This construct was used as bait to identify TRAF3-interacting proteins encoded by a cDNA library constructed from an EBV-transformed lymphoblastoid cell line (a gift of S. Elledge, Baylor College of Medicine).

### Immunoprecipitation and immunoblot analysis

Immunoprecipitation assays were performed as previously described [29]. Briefly, HEK293FT cells were co-transfected with vectors expressing a His-tagged GMEB1 protein (GMEB1-His) (1 μg) together with a FLAG-tagged TRAF3 protein (TRAF3 wt-flag) (0.5 μg) or the FLAG-tagged mutant forms of TRAF3, TRAF3 M7 (TRAF3 M7-flag) (0.5 μg) or TRAF3ΔC-flag (0.5 μg), using the calcium phosphate transfection method. The cells were harvested approximately 20 h post transfection and lysed in 0.5% NP40 lysis buffer [25 mM Tris–HCL pH 7.4, 150 mM NaCl, 5 mM EDTA, 1% Glycerol, 0.5% NP-40, 1 mM DTT, 1 mM PMSF 100 mM, 1 × protease inhibitor cocktail (Roche, Basel, Switzerland)]. His-tagged proteins (GMEB1-His) were immunoprecipitated with Ni–NTA beads (Qiagen, Hilden, Germany). The immunoprecipitated complex (IP) or total cell lysates were analyzed by SDS-PAGE on a 7.5–8% gel and subjected to immunoblot analysis with the anti-His rabbit polyclonal antibody His probe (Santa Cruz Biotechnology, Santa Cruz, USA), recognizing GMEB1 (80 kDa) or the anti-FLAG monoclonal antibody M5 (Sigma-Aldrich, St. Louis, USA), recognizing both TRAF3 wt (58 kDa) and TRAF3 M7 (58 kDa). The LMP1 monoclonal antibody OT22CN, which recognizes the amino-terminus of LMP1, was used against LMP1(1–231), and the anti-β-Actin (Santa Cruz Biotechnology, Santa Cruz, USA) was used against β-actin.

### RNA interference

The expression of TRAF3 was downregulated by RNA interference, as previously described [29]. For this purpose, HEK293 cells were transfected with a shRNA-expression vector targeting TRAF3 (TRAF3shRNA), or a control shRNAluc, in the presence or absence of GMEB1-His expression vector.

### MTT viability assays

MTT viability assays were performed using 3-(4,5-dimethylthiazol-2-yl)-2,5-diphenyltetrazolium bromide (MTT) (Sigma-Aldrich), as previously described [30]. Briefly, HeLa cells were seeded at a density of 4 × 10^5^ per well in a 6-well plate and transfected with the indicated expression vectors. 20 h post transfection, HeLa cells were trypsinized, seeded at a density of 5 × 10^3^ cells per well in 96 well flat bottom culture plates, and treated with cycloheximide (CHX) (20 μg ml^−1^) and TNF-α (10 ng ml^−1^) for 24 h, in order to induce apoptosis. After treatment, MTT was added to a final concentration of 0.45 mg ml^−1^ in each well (final volume 220 μl). The culture plate was incubated for 4 h at 37 °C with 5% v/v CO_2_ level. In order to dissolve insoluble formazan crystals formed in mitochondria, 150 μl of acid isopropanol containing 10% Triton-X (Applichem, Darmstadt, Germany) were added to each well and the plate was incubated under shaking for 5 min. The absorbance of the resulting purple color was measured at 570 nm, using a Microplate Autoreader (Biotek, Winooski, VT, USA). Each sample was tested in triplicates and experiments were repeated three or four times.

### Statistical analysis

All experiments were performed in triplicates and analysed by Student’s t-test (*p* < 0.05). Numerical data were expressed as mean ± Standard Error of the Mean (SEM). Statistical analysis was performed using the software SPSS ver. 22.0 (IBM Corp., Armonk, NY, USA).

## Data Availability

All data generated by this study are included in the manuscript, all materials are available.

## Abbreviations

TNF: Tumor Necrosis Factor
TRAFs: TNF Receptor-Associated Factors
GMEB1: Glucocorticoid Modulatory Element-Binding Protein 1
HPV: Human papilloma virus
HNSCC: Head and neck squamous cell carcinoma
HSV: Herpes Simplex Virus
EBV: Epstein Barr virus
CHX: Cycloheximide
